# Supplementary material for: Do patients’ characteristics influence their healthcare concerns?—A hospital care survey
Source: PLoS One. 2021 Oct 14;16(10):e0258618. doi: 10.1371/journal.pone.0258618 (PMC8516281; doi:10.1371/journal.pone.0258618)
Supplement: S1 Appendix — (DOCX) [file pone.0258618.s001.docx]

| Service in the hospital that took care of patient |
| --- |
| Family medicine |
| Internal medicine |
| Orthopedic surgery |
| General surgery |
| Cardiology |
| Oncology |
| Transplant surgery |
| Cardiothoracic surgery |
| Other (specify) |
| Age and Gender |
| Educational level |
| Some high school, but did not graduate |
| High school graduate or GED |
| Some college or 2-year degree |
| 4-year college graduate |
| More than 4-year college degree |
| Are you of Spanish/Hispanic or Latino origin or descent? |
| Not Spanish/Hispanic/Latino |
| Puerto Rican |
| Mexican/Mexican American/Chicano |
| Cuban |
| Other Spanish/Hispanic/Latino |
| Ethnicity (White, Black or African American, Asian, American Indian or Alaska Native) |
| Main language spoke at home (English, Spanish, Chinese, Other) |
| Select the items you consider very important and which are not important for you in a hospital: |
| 1. How clean my room and bathroom were |
| 1. My risk of falling while I am in the hospital |
| 1. My risk of getting an infection while I am in the hospital |
| 1. How likely I will have to come back once I leave the hospital |
| 1. How long I will need to stay in the hospital. |
| 1. How often the area around my room was quiet at night |
| 1. The doctors explaining things in a way that you could understand |
| 1. The doctors listening carefully to you. |
| 1. The doctors treating you with courtesy and respect |
| Have looked up the hospital on the internet? Which website or journal: |
| Consumer Reports, Google, Healthgrades, Medicare/Hospital Compare, US News and World Report, Other |
| Regarding hospital measures for improvement. How would you prefer to view them? |
| Line graph, Bar chart, Pie, List, Other (specify) |
