# Supplementary material for: Do patients’ characteristics influence their healthcare concerns?—A hospital care survey
Source: PLoS One. 2021 Oct 14;16(10):e0258618. doi: 10.1371/journal.pone.0258618 (PMC8516281; doi:10.1371/journal.pone.0258618)
Supplement: S2 Appendix — (DOCX) [file pone.0258618.s002.docx]

**Results**

Table 1. Data summary for all the patients.

Table 2. Survey answers by age

Table 3. Survey answers by gender

Table 4. Survey answers by education

Table 5. Dichotomized survey answers (“very important” vs not) by age

Table 6. Dichotomized survey answers (“very important” vs not) by gender

Table 7. Dichotomized survey answers (“very important” vs not) by education

Table 1.

|  | **All patients (N=262)** |
| --- | --- |
| Service in the hospital that took care of patient (select all that apply) |  |
| Family Medicine | 106 (40.5%) |
| Internal Medicine | 90 (34.4%) |
| Orthopedic Surgery | 15 (5.7%) |
| General Surgery | 15 (5.7%) |
| Cardiology | 13 (5.0%) |
| Oncology | 4 (1.5%) |
| Transplant Surgery | 2 (0.8%) |
| Cardiothoracic Surgery | 2 (0.8%) |
| Other | 17 (6.5%) |
| Other service |  |
| Cardiothoracic Surgery | 1 (5.9%) |
| Gynecology | 2 (11.8%) |
| Neuology | 1 (5.9%) |
| Neurology | 2 (11.8%) |
| Neurosurgery | 1 (5.9%) |
| OB/GYN | 1 (5.9%) |
| Ophthalmology | 1 (5.9%) |
| Plastic Surgery | 1 (5.9%) |
| Transplant | 1 (5.9%) |
| UROGYNECOLOGY | 1 (5.9%) |
| Urologic  Surgery | 1 (5.9%) |
| Urology | 2 (11.8%) |
| Vascular Surgery | 1 (5.9%) |
| urogynecology | 1 (5.9%) |
| Age | 70 (20.0-95.0) |
| Gender |  |
| Male | 129 (49.2%) |
| Female | 133 (50.8%) |
| What is the highest grade or level of school that you have completed? |  |
| Some high school, but did not graduate | 6 (2.3%) |
| High school graduate or GED | 34 (13.0%) |
| Some college or 2-year degree | 87 (33.2%) |
| 4-year college graduate | 64 (24.4%) |
| More than 4-year college degree | 71 (27.1%) |
| Are you of Spanish/Hispanic or Latino origin or descent? |  |
| Missing | 4 |
| Not Spanish/Hispanic/Latino | 247 (95.7%) |
| Puerto Rican | 5 (1.9%) |
| Mexican/Mexican American/Chicano | 2 (0.8%) |
| Cuban | 1 (0.4%) |
| Other Spanish/Hispanic/Latino | 3 (1.2%) |
| Race |  |
| White | 230 (87.8%) |
| Black or African American | 22 (8.4%) |
| Asian | 6 (2.3%) |
| American Indian or Alaska Native | 1 (0.4%) |
| What language do you mainly speak at home? |  |
| English | 257 (98.1%) |
| Spanish | 2 (0.8%) |
| Chinese | 1 (0.4%) |
| Other | 3 (1.1%) |
| Other language |  |
| ALBENIAN | 1 (33.3%) |
| Danish | 1 (33.3%) |
| Filipino | 1 (33.3%) |
| Grade the folllowing from Very important to Not important to you: |  |
| How clean my room and bathroom were. |  |
| Missing | 1 |
| Very important | 114 (43.7%) |
| Not important | 147 (56.3%) |
| My risk of falling while I am in the hospital. |  |
| Very important | 40 (15.3%) |
| Not important | 222 (84.7%) |
| My risk of getting an infection while I am in the hospital. |  |
| Very important | 176 (67.2%) |
| Not important | 86 (32.8%) |
| How likely I will have to come back once I leave the hospital. |  |
| Missing | 1 |
| Very important | 78 (29.9%) |
| Not important | 183 (70.1%) |
| How long I will need to stay in the hospital. |  |
| Very important | 143 (54.6%) |
| Not important | 119 (45.4%) |
| How often the area around your room was quiet at night. |  |
| Missing | 2 |
| Very important | 98 (37.7%) |
| Not important | 162 (62.3%) |
| The doctors explaining things in a way that you could understand. |  |
| Missing | 2 |
| Very important | 168 (64.6%) |
| Not important | 92 (35.4%) |
| The doctors listening carefully to you. |  |
| Missing | 1 |
| Very important | 154 (59.0%) |
| Not important | 95 (41.0%) |
| The doctors treating you with courtesy and respect. |  |
| Missing | 1 |
| Very important | 113 (43.3%) |
| Not important | 148 (56.7%) |
| Have looked up a hospital on the internet | 49 (18.7%) |
| Which site: |  |
| Consumer report | 17 (6.5%) |
| Google | 25 (9.5%) |
| Health Grades | 18 (6.9%) |
| Hospital Compare | 13 (5.0%) |
| US News and World Report | 19 (7.3%) |
| Other | 1 (0.4%) |
| Other rating site |  |
| NIH | 1 (100.0%) |
| We want to share our hospital measures for improvement. How would you prefer to view them? | |
| A line graph | 48 (18.3%) |
| A bar chat | 104 (39.7%) |
| A pie chat | 38 (14.5%) |
| A list | 168 (64.1%) |
| Other | 0 |

Table 2.

|  | **Less than 65 years old (N=93)** | **65 years or older (N=169)** | **P-value** |
| --- | --- | --- | --- |
| How clean my room and bathroom were. |  |  | 0.13 |
| Missing | 0 | 1 |  |
| Very important | 34 (36.6%) | 80 (47.6%) |  |
| Not important | 59 (63.4%) | 88 (52.4%) |  |
| My risk of falling while I am in the hospital. |  |  | 0.29 |
| Very important | 10 (10.8%) | 30 (17.8%) |  |
| Not important | 83 (89.2%) | 139 (82.2%) |  |
| My risk of getting an infection while I am in the hospital. |  |  | 0.38 |
| Very important | 66 (71.0%) | 110 (65.1%) |  |
| Not important | 27 (29.0%) | 59 (34.9%) |  |
| How likely I will have to come back once I leave the hospital. |  |  | 0.12 |
| Missing | 0 | 1 |  |
| Very important | 28 (30.1%) | 50 (29.8%) |  |
| Not important | 65 (69.9%) | 118 (70.2%) |  |
| How long I will need to stay in the hospital. |  |  | 0.24 |
| Very important | 55 (59.1%) | 88 (52.0%) |  |
| Not important | 38 (40.9%) | 81 (48.0%) |  |
| How often the area around your room was quiet at night. |  |  | 0.16 |
| Missing | 0 | 2 |  |
| Very important | 28 (30.1%) | 70 (41.9%) |  |
| Not important | 65 (69.9%) | 97 (58.1%) |  |
| The doctors explaining things in a way that you could understand. | |  | 0.054 |
| Missing | 1 | 1 |  |
| Very important | 51 (55.4%) | 117 (69.6%) |  |
| Not important | 41 (44.6%) | 51 (30.3%) |  |
| The doctors listening carefully to you. |  |  | 0.30 |
| Missing | 0 | 1 |  |
| Very important | 49 (52.7%) | 105 (62.5%) |  |
| Not important | 44 (47.3%) | 63 (37.5%) |  |
| The doctors treating you with courtesy and respect. |  |  | 0.37 |
| Missing | 0 | 1 |  |
| Very important | 35 (37.6%) | 78 (46.4%) |  |
| Not important | 58 (62.4%) | 90 (53.6%) |  |
| Have looked up a hospital on the internet | 21 (22.6%) | 28 (16.6%) | 0.23 |
| Which site: |  |  |  |
| Consumer report | 7 (7.5%) | 10 (5.9%) | 0.61 |
| Google | 11 (11.8%) | 14 (8.3%) | 0.35 |
| Health Grades | 11 (11.8%) | 7 (4.1%) | 0.019 |
| Hospital Compare | 2 (2.2%) | 11 (6.5%) | 0.12 |
| US News and World Report | 12 (12.9%) | 7 (4.1%) | 0.009 |
| Other | 0 (0.0%) | 1 (0.6%) | 0.46 |
| Other rating site |  |  |  |
| NIH | 0 (0.0%) | 1 (100.0%) |  |
| We want to share our hospital measures for improvement. How would you prefer to view them? | | |  |
| A line graph | 16 (17.2%) | 32 (18.9%) | 0.73 |
| A bar chat | 38 (40.9%) | 66 (39.1%) | 0.77 |
| A pie chat | 15 (16.1%) | 23 (13.6%) | 0.58 |
| A list | 58 (62.4%) | 110 (65.1%) | 0.66 |
| Other | 0 | 0 |  |

Table 3.

|  | **Male (N=129)** | **Female (N=133)** | **P-value** |
| --- | --- | --- | --- |
| How clean my room and bathroom were. |  |  | 0.78 |
| Missing | 0 | 1 |  |
| Very important | 59 (45.7%) | 55 (41.7%) |  |
| Not important | 70 (54.3%) | 77 (58.3%) |  |
| My risk of falling while I am in the hospital. |  |  | 0.009 |
| Very important | 13 (10.1%) | 27 (20.3%) |  |
| Not important | 116 (89.9%) | 106 (79.7%) |  |
| My risk of getting an infection while I am in the hospital. |  |  | 0.96 |
| Very important | 86 (66.7%) | 90 (67.7%) |  |
| Not important | 43 (33.4%) | 43 (32.4%) |  |
| How likely I will have to come back once I leave the hospital. |  |  | 0.71 |
| Missing | 0 | 1 |  |
| Very important | 39 (30.2%) | 39 (29.5%) |  |
| Not important | 90 (69.8%) | 93 (70.5%) |  |
| How long I will need to stay in the hospital. |  |  | 0.83 |
| Very important | 72 (55.8%) | 71 (53.4%) |  |
| Not important | 57 (44.2%) | 62 (46.6%) |  |
| How often the area around your room was quiet at night. |  |  | 0.65 |
| Missing | 0 | 2 |  |
| Very important | 52 (40.3%) | 46 (35.1%) |  |
| Not important | 77 (59.7%) | 85 (64.9%) |  |
| The doctors explaining things in a way that you could understand. | |  | 0.47 |
| Missing | 0 | 2 |  |
| Very important | 88 (68.2%) | 80 (61.1%) |  |
| Not important | 41 (31.8%) | 51 (38.9%) |  |
| The doctors listening carefully to you. |  |  | 0.30 |
| Missing | 0 | 1 |  |
| Very important | 70 (54.3%) | 84 (63.6%) |  |
| Not important | 59 (45.7%) | 48 (36.4%) |  |
| The doctors treating you with courtesy and respect. |  |  | 0.37 |
| Missing | 0 | 1 |  |
| Very important | 56 (43.4%) | 57 (43.2%) |  |
| Not important | 73 (56.6%) | 75 (56.8%) |  |
| Have looked up a hospital on the internet | 22 (17.1%) | 27 (20.3%) | 0.50 |
| Which site: |  |  |  |
| Consumer report | 6 (4.7%) | 11 (8.3%) | 0.23 |
| Google | 13 (10.1%) | 12 (9.0%) | 0.77 |
| Health Grades | 7 (5.4%) | 11 (8.3%) | 0.36 |
| Hospital Compare | 6 (4.7%) | 7 (5.3%) | 0.82 |
| US News and World Report | 6 (4.7%) | 13 (9.8%) | 0.11 |
| Other | 0 (0.0%) | 1 (0.8%) | 0.32 |
| Other rating site |  |  |  |
| NIH | 0 (0.0%) | 1 (100.0%) |  |
| We want to share our hospital measures for improvement. How would you prefer to view them? | | | |
| A line graph | 30 (23.3%) | 18 (13.5%) | 0.042 |
| A bar chat | 55 (42.6%) | 49 (36.8%) | 0.34 |
| A pie chat | 18 (14.0%) | 20 (15.0%) | 0.80 |
| A list | 81 (62.8%) | 87 (65.4%) | 0.66 |
| Other | 0 | 0 |  |

Table 4.

|  | **Less than 4 years of college (N=127)** | **4 or more years of college (N=135)** | **P-value** |
| --- | --- | --- | --- |
| How clean my room and bathroom were. |  |  | 0.14 |
| Missing | 1 | 0 |  |
| Very important | 63 (50.0%) | 51 (37.8%) |  |
| Not important | 63 (50.0%) | 84 (62.2%) |  |
| My risk of falling while I am in the hospital. |  |  | 0.15 |
| Very important | 25 (19.7%) | 15 (11.1%) |  |
| Not important | 102 (80.3%) | 120 (88.9%) |  |
| My risk of getting an infection while I am in the hospital. |  |  | 0.12 |
| Very important | 78 (61.4%) | 98 (72.6%) |  |
| Not important | 49 (38.6%) | 37 (27.4%) |  |
| How likely I will have to come back once I leave the hospital. |  |  | 0.83 |
| Missing | 1 | 0 |  |
| Very important | 39 (31.0%) | 39 (28.9%) |  |
| Not important | 87 (69.0%) | 96 (71.1%) |  |
| How long I will need to stay in the hospital. |  |  | 0.98 |
| Very important | 69 (54.3%) | 74 (54.8%) |  |
| Not important | 58 (45.7%) | 61 (45.2%) |  |
| How often the area around your room was quiet at night. |  |  | 0.20 |
| Missing | 1 | 1 |  |
| Very important | 52 (41.3%) | 46 (34.3%) |  |
| Not important | 74 (58.7%) | 88 (65.6%) |  |
| The doctors explaining things in a way that you could understand. |  |  | 0.33 |
| Missing | 1 | 1 |  |
| Very important | 85 (67.5%) | 83 (61.9%) |  |
| Not important | 41 (32.5%) | 51 (38.0%) |  |
| The doctors listening carefully to you. |  |  | 0.17 |
| Missing | 0 | 1 |  |
| Very important | 82 (64.6%) | 72 (53.7%) |  |
| Not important | 45 (35.4%) | 62 (46.3%) |  |
| The doctors treating you with courtesy and respect. |  |  | 0.001 |
| Missing | 1 | 0 |  |
| Very important | 67 (53.2%) | 46 (34.1%) |  |
| Not important | 59 (46.8%) | 89 (65.9%) |  |
| Have looked up a hospital on the internet | 19 (15.0%) | 30 (22.2%) | 0.13 |
| Which site: |  |  |  |
| Consumer report | 9 (7.1%) | 8 (5.9%) | 0.70 |
| Google | 10 (7.9%) | 15 (11.1%) | 0.37 |
| Health Grades | 6 (4.7%) | 12 (8.9%) | 0.18 |
| Hospital Compare | 2 (1.6%) | 11 (8.1%) | 0.014 |
| US News and World Report | 6 (4.7%) | 13 (9.6%) | 0.13 |
| Other | 0 (0.0%) | 1 (0.7%) | 0.33 |
| Other rating site |  |  |  |
| NIH | 0 (0.0%) | 1 (100.0%) |  |
| We want to share our hospital measures for improvement. How would you prefer to view them? | | |  |
| A line graph | 14 (11.0%) | 34 (25.2%) | 0.003 |
| A bar chat | 41 (32.3%) | 63 (46.7%) | 0.017 |
| A pie chat | 15 (11.8%) | 23 (17.0%) | 0.23 |
| A list | 83 (65.4%) | 85 (63.0%) | 0.69 |
| Other | 0 | 0 |  |

Table 5.

|  | **Less than 65 years old (N=93)** | **65 years or older (N=169)** | **P-value** |
| --- | --- | --- | --- |
|  |  |  |  |
| How clean my room and bathroom were. | 34 (36.6%) | 80 (47.6%) | 0.085 |
| My risk of falling while I am in the hospital. | 10 (10.8%) | 30 (17.8%) | 0.13 |
| My risk of getting an infection while I am in the hospital. | 66 (71.0%) | 110 (65.1%) | 0.33 |
| How likely I will have to come back once I leave the hospital. | 28 (30.1%) | 50 (29.8%) | 0.95 |
| How long I will need to stay in the hospital. | 55 (59.1%) | 88 (52.1%) | 0.27 |
| **How often the area around your room was quiet at night.** | **28 (30.1%)** | **70 (41.9%)** | **0.06** |
| **The doctors explaining things in a way that you could understand.** | **51 (55.4%)** | **117 (69.6%)** | **0.022** |
| The doctors listening carefully to you. | 49 (52.7%) | 105 (62.5%) | 0.12 |
| The doctors treating you with courtesy and respect. | 35 (37.6%) | 78 (46.4%) | 0.17 |

Table 6.

|  | **Male (N=129)** | **Female (N=133)** | **P-value** |
| --- | --- | --- | --- |
| How clean my room and bathroom were. | 59 (45.7%) | 55 (41.7%) | 0.51 |
| **My risk of falling while I am in the hospital.** | **13 (10.1%)** | **27 (20.3%)** | **0.021** |
| My risk of getting an infection while I am in the hospital. | 86 (66.7%) | 90 (67.7%) | 0.86 |
| How likely I will have to come back once I leave the hospital. | 39 (30.2%) | 39 (29.5%) | 0.9 |
| How long I will need to stay in the hospital. | 72 (55.8%) | 71 (53.4%) | 0.69 |
| How often the area around your room was quiet at night. | 52 (40.3%) | 46 (35.1%) | 0.39 |
| The doctors explaining things in a way that you could understand. | 88 (68.2%) | 80 (61.1%) | 0.23 |
| The doctors listening carefully to you. | 70 (54.3%) | 84 (63.6%) | 0.12 |
| The doctors treating you with courtesy and respect. | 56 (43.4%) | 57 (43.2%) | 0.97 |

Table 7.

|  | **Less than 4 years of college (N=127)** | **4 or more years of college (N=135)** | **P-value** |
| --- | --- | --- | --- |
| **How clean my room and bathroom were.** | **63 (50.0%)** | **51 (37.8%)** | **0.047** |
| My risk of falling while I am in the hospital. | 25 (19.7%) | 15 (11.1%) | 0.054 |
| My risk of getting an infection while I am in the hospital. | 78 (61.4%) | 98 (72.6%) | 0.054 |
| How likely I will have to come back once I leave the hospital. | 39 (31.0%) | 39 (28.9%) | 0.72 |
| How long I will need to stay in the hospital. | 69 (54.3%) | 74 (54.8%) | 0.94 |
| How often the area around your room was quiet at night. | 52 (41.3%) | 46 (34.3%) | 0.25 |
| The doctors explaining things in a way that you could understand. | 85 (67.5%) | 83 (61.9%) | 0.35 |
| The doctors listening carefully to you. | 82 (64.6%) | 72 (53.7%) | 0.075 |
| **The doctors treating you with courtesy and respect.** | **67 (53.2%)** | **46 (34.1%)** | **0.002** |
