# Supplementary material for: Do patients’ characteristics influence their healthcare concerns?—A hospital care survey
Source: PLoS One. 2021 Oct 14;16(10):e0258618. doi: 10.1371/journal.pone.0258618 (PMC8516281; doi:10.1371/journal.pone.0258618)
Supplement: Attachment — Submitted filename: Response to Reviewers.doc [file pone.0258618.s003.doc]

Miss Seema Biswas

Sharon Mary Brownie

Academic Editor

PLOS ONE

**Re: Manuscript Revision**

Dear Sharon Mary Brownie,

Please find, attached, a revised version of the manuscript entitled “Do Patients’ Characteristics Influence Their Healthcare Concerns? – A Hospital Care Survey,” The authors’ responses to reviewers’ suggestions below are in bold italic font, and the requested edits are highlighted by underlining the edited text in the revised manuscript.

On behalf of the authors, I would like to express our sincere appreciation for the reviewers’ feedback and suggestions. We sincerely hope that this revised manuscript is now suitable for publication at PLOS ONE. Please feel free to contact me if you have any additional suggestions or concerns regarding the manuscript.

Sincerely,

Tais Garcia de Oliveira Bertasi, MD

Mayo Clinic

Jacksonville, FL

**Reviewer's Responses to Questions**

**1. Is the manuscript technically sound, and do the data support the conclusions?**

The manuscript must describe a technically sound piece of scientific research with data that supports the conclusions. Experiments must have been conducted rigorously, with appropriate controls, replication, and sample sizes. The conclusions must be drawn appropriately based on the data presented.

**Reviewer #1:** Yes

**Reviewer #2:** Partly

**Reviewer #3:** Partly

**2. Has the statistical analysis been performed appropriately and rigorously?**

**Reviewer #1:** Yes

**Reviewer #2:** I Don't Know

**Reviewer #3:** Yes

**3. Have the authors made all data underlying the findings in their manuscript fully available?**

The PLOS Data policy requires authors to make all data underlying the findings described in their manuscript fully available without restriction, with rare exception (please refer to the Data Availability Statement in the manuscript PDF file). The data should be provided as part of the manuscript or its supporting information, or deposited to a public repository. For example, in addition to summary statistics, the data points behind means, medians and variance measures should be available. If there are restrictions on publicly sharing data—e.g. participant privacy or use of data from a third party—those must be specified.

**Reviewer #1:** Yes

**Reviewer #2:** No

**Reviewer #3:** No

**4. Is the manuscript presented in an intelligible fashion and written in standard English?**

PLOS ONE does not copyedit accepted manuscripts, so the language in submitted articles must be clear, correct, and unambiguous. Any typographical or grammatical errors should be corrected at revision, so please note any specific errors here.

**Reviewer #1:** Yes

**Reviewer #2:** Yes

**Reviewer #3:** Yes

***The authors appreciate the reviewers’ availability and great suggestions. We made several edits to the manuscript and included Appendix 2 with the raw data collected with the survey answers.***

**Reviewer's Comments to the Author**

**REVIEWER 1:**

**1.** If a linelist of the potential samples were made to select study samples at random.
***The authors appreciate your comment. The randomization process was included in the Methods section and is described below in the answer for the 1^st^ comment from Reviewer 2.***

**2.** Could all the tables and figures have a title on the top of each.

***Thank you for your suggestion. We included a title on the top of the figures and table.***

**3.** Could there be chance of recall bias particularly for aged respondents while the interview was done 50 or more days after discharge from the hospital.

***The authors appreciate your comment. We have indicated this possibility in the limitations section.***

**4.** The demographic information table (table 1) could contain information of distribution of respondents by gender and age group.

***Thank you for your suggestion. We added the age and sex information on Table 1.***

**REVIEWER 2:**

**1.** It is not possible to interpret the findings in the absence of information on the randomization process and the response rate to the survey, both overall and in terms of key characteristics of the respondents

***The authors appreciate your comment. The randomization process was included in the Methods section. The authors of the study were the investigators who approached patients in waiting rooms in all the Mayo buildings, in hospital rooms, and patient rooms, depending on the setting they are in, at various days of the study period. They asked if patients were recently hospitalized if they were in the waiting room or patient room. A total of 142 patients declined participating in the study, with a response rate of 64.8%. Consent was obtained for those interested and study survey was given.***

***Participants were approached randomly to maximize experience breadth. For example, if only patients in one building were approached, only patients admitted into specific services (e.g. Family Medicine, Internal Medicine, Rehabilitation Medicine) would be surveyed. The authors wanted to cover all experiences from admission into all services offered in the hospital, as much as possible and hoped that by spreading out in this manner, there would be good representation of all types of patient admission.***

**REVIEWER 3:**

This is an interesting study, but there are some concerns that need to be addressed.

**1.** After reading the introduction, I was unsure whether the authors disagree with using the CAHPS for reporting hospital quality or not. Although the CAHPS/CMS aggregates measures for hospital reporting (e.g., hospital compare), researchers could request the data to understand more granular level information and patterns (e.g., patient characteristics). Link: https://hcahpsonline.org/globalassets/hcahps/survey-instruments/mail/qag-v16.0-materials/2021_survey-instruments_english_mail.pdf

***Thank you for your comment and for the link. The authors agree that it was not clear whether we disagree with using the CAHPS for reporting hospital quality. Therefore, we included some sentences at the end of the Introduction section to make it clearer and to explain that the aim of our study is to understand in more detailed the patients’ concerns behind each response to those surveys.***

**2.** I would encourage the authors to review sections of the introduction. For example: “Some factors that affect patient survey scores may not be directly related to hospital performance. [5] Characteristics of different patient populations may make certain parts of the survey more relevant than others. Hospitals may have very little control on the education level and age of patients who present for care. It is known that more educated and younger patients tend to evaluate health care less positively.” I would hope that hospitals “want” to treat everyone equally. Surveys are designed to get a range of responses that would be representative of the overall population.

***The authors appreciate your comment. We have changed the introduction to make our message clearer.***

**3.** The authors mentioned “A survey developed via literature review and specialist recommendations was applied in different departments of our tertiary care center during the year of 2016.” More information about how the survey questions were developed is needed. What article(s) or literature was used to develop the questions?

***Thank you for your suggestion. We included the references of the literature used to develop the survey.***

**4.** It is unclear to me why the patients needed to be recently admitted to the hospital or admitted more than 50 days before the survey was being applied. Explaining this range may be important. In addition, what does it mean to be recently admitted?

***Thank you for your comment. Recently admitted means admitted within 1-2 weeks. The survey from Mayo Clinic usually arrives more than 50 days after patients are admitted. The reason for this is unclear but may be because surveyors want to determine what remains in patients’ recollections after such a period of time has elapsed.***

**5.** More details are needed about “The investigators randomly approached the patients in different hospital departments to solicit their participation.” Are these the authors in the paper, RAs, others? How many investigators were conducting the surveys? Why were participants approached randomly? I would assume that the investigators were hoping for some representation. How many people declined to participate?

***The authors of the study were the investigators who approached patients in waiting rooms in all the Mayo buildings, in hospital rooms, and patient rooms, depending on the setting they are in, at various days of the study period. They asked if patients were recently hospitalized if they were in the waiting room or patient room. Consent was obtained for those interested and study survey was given.***

***Participants were approached randomly to maximize experience breadth. For example, if only patients in one building were approached, only patients admitted into specific services (e.g. Family Medicine, Internal Medicine, Rehabilitation Medicine) would be surveyed. The authors wanted to cover all experiences from admission into all services offered in the hospital, as much as possible and hoped that by spreading out in this manner, there would be good representation of all types of patient admission. A sentence was included in the Methods section to make it clearer. A total of 142 patients declined participating in the study (the response rate was included in the Results section)***

**6.** In the discussion, the authors mentioned that “‘Acquiring an infection while receiving health care is also known as “health care-associated Infections” (HAI) [11]’” They may need to expand about how common these infections are and provide some explanations as to why they believe this was the top concern.

***Thank you for your suggestion. The authors included a few sentences in the Discussion section about the frequency of HAIs and why we believe this was a top concern for the patients.***

**7.** The authors also mentioned that “physicians conducted the surveys, which may have influenced the responses.” How do the authors believe that this may have influenced the responses?

***The authors believe that having physicians conducting the surveys may have influenced who they gave surveys to. For example, physicians may not want to give the surveys to patients they took care of. However, the authors/investigators tried not to be affected by this factor when giving out the surveys.***

**8.** Finally, these data are somewhat dated. The authors may need to include a paragraph in the discussion about how the current epidemic may have changed/influences or make this survey more relevant.

***The authors appreciate this interesting comment. We included a comment about this in the discussion section.***
