# Supplementary material for: Do patients’ characteristics influence their healthcare concerns?—A hospital care survey
Source: PLoS One. 2021 Oct 14;16(10):e0258618. doi: 10.1371/journal.pone.0258618 (PMC8516281; doi:10.1371/journal.pone.0258618)
Supplement: Attachment — Submitted filename: Response to Reviewers.doc [file pone.0258618.s004.doc]

Miss Seema Biswas

Sharon Mary Brownie

Academic Editor

PLOS ONE

**Re: Manuscript Revision**

Dear Sharon Mary Brownie,

Please find, attached, a revised version of the manuscript entitled “Do Patients’ Characteristics Influence Their Healthcare Concerns? – A Hospital Care Survey.” The authors’ responses to reviewers’ suggestions below are in bold italic font, and the requested edits are highlighted in red in the revised manuscript.

On behalf of the authors, I would like to express our sincere appreciation for the reviewers’ feedback and suggestions. We sincerely hope that this revised manuscript is now suitable for publication at PLOS ONE. Please feel free to contact me if you have any additional suggestions or concerns regarding the manuscript.

Sincerely,

Tais Garcia de Oliveira Bertasi, MD

Mayo Clinic

Jacksonville, FL

**Reviewer's Comments to the Author**

REVIEWER #1:

The respondent selection criteria regarding hospital admission as mentioned, "recently admitted or admitted more the 50 days before the survey was applied" is not clear. If data was collected from patients admitted a week before or 90 days before data collection, should it not be a range (e.g. admitted 10 - 50 days before data collection)?

**The authors agree with your comment. The methodology section was rewritten in agreement with your recommendation.**

REVIEWER #2:

This version does not address my previous comments. Simply stating that patients were approached randomly does not adequately describe the randomisation process. Further, the response rate according to key characteristics is not provided. It could be that the response rate varied significantly by race, education, age gender, etc., so introducing potential biases. This information is required for a determination of the validity of the findings.

**The authors appreciate your comment. The methodology section was rewritten to address the previous comment. Regarding the response rate, as it is possible to see on the appendix showing all data, the maximum of missing answers for each of the nine questionnaire questions were from two participants; this fortunately would not make a statistically significant difference when accounting for race, education, age, and gender. However, the reviewer made a great point, and the potential bias is now included in the limitations section.**
